# Hyaluronic Acid-Based Microparticles with Lubrication and Anti-Inflammation for Alleviating Temporomandibular Joint Osteoarthritis

**DOI:** 10.34133/bmr.0073

**Published:** 2024-09-06

**Authors:** Lei Liu, Gang He, Yixi Li, Yiwen Xian, Guixian He, Yonglong Hong, Chong Zhang, Decheng Wu

**Affiliations:** ^1^ Guangdong Provincial Key Laboratory of Advanced Biomaterials, Department of Biomedical Engineering, Southern University of Science and Technology, Shenzhen 518055, China.; ^2^ Department of Maxillofacial Surgery, Shenzhen Hospital, Southern Medical University, Shenzhen 518101, China.

## Abstract

The pathogenesis of temporomandibular joint osteoarthritis (TMJOA) is closely associated with mechanical friction, which leads to the up-regulation of inflammatory mediators and the degradation of articular cartilage. Injectable drug-loaded microparticles have attracted widespread interest in intra-articular treatment of TMJOA by providing lubrication and facilitating localized drug delivery. Herein, a hyaluronic acid-based microparticle is developed with excellent lubrication properties, drug loading capacity, antioxidant activity, and anti-inflammatory effect for the treatment of TMJOA. The microparticles are facilely prepared by the self-assembly of 3-aminophenylboronic acid-modified hyaluronic acid (HP) through hydrophobic interaction in an aqueous solution, which can further encapsulate diol-containing drugs through dynamic boronate ester bonds. The resulting microparticles demonstrate excellent injectability, lubrication properties, radical scavenging efficiency, and antibacterial activity. Additionally, the drug-loaded microparticles exhibit a favorable cytoprotective effect on chondrocyte cells in vitro under an oxidative stress microenvironment. In vivo experiments validate that intra-articular injection of drug-loaded microparticles effectively alleviates osteoporosis-like damage, suppresses inflammatory response, and facilitates matrix regeneration in the treatment of TMJOA. The HP microparticles demonstrate excellent injectability and encapsulation capacity for diol-containing drugs, highlighting its potential as a versatile drug delivery vehicle in the intra-articular treatment of TMJOA.

## Introduction

Temporomandibular joint (TMJ) is located between the mandibular condyle and temporal bone on both sides of the face [1]. As one of the diarthrodial synovial joints, TMJ plays a crucial role in facilitating various oral and mandibular movements, including articulation, mastication, suction, swallowing, and facial expressions [2]. An epidemiological study reveals that approximately 60% to 70% of adults experience symptoms associated with TMJ disorder [3], among whom about 8% to 16% suffer from TMJ osteoarthritis (TMJOA) [4]. TMJOA represents the most severe form of TMJ disorder [5], characterized by symptoms such as pain, restricted mandibular movement, TMJ clicking, and muscle spasms that seriously affect patients’ life quality. Unlike other forms of OA, TMJOA can occur at an early stage in life, even during adolescence. Moreover, the prevalence of TMJOA increases with age [6]. Nowadays, TMJOA is widely recognized as a chronic degenerative disorder characterized by persistent inflammation and gradual degradation of the articular cartilage [4]. However, the underlying pathogenesis of TMJOA remains unclear, making it difficult to develop radical treatments [7]. Therefore, interventions aimed at minimizing inflammation and preventing joint degeneration are currently the preferred strategies for managing TMJOA.

The strategies for treating OA can be categorized into noninvasive, minimally invasive, and surgical interventions [8]. However, the controversial effectiveness of physical therapy, potential gastrointestinal toxicity associated with oral medications, and surgical-induced tissue damage have raised considerable concerns [9]. Thus, considerable interest has been given to the local administration of pharmacological agents through intra-articular injection, due to its unique advantages in enhancing the bioavailability of therapeutics, reducing systemic toxicity, facilitating ease of use, and enabling minimally invasive manipulation [10]. As TMJ undergoes the most complex 3-dimensional movements among all synovial joints in the human body [11,12], TMJOA is widely recognized as a typical disease strongly associated with friction. Excessive mechanical loading can initiate cartilage wear, which exacerbates the inflammatory response and accelerates TMJOA progression [13]. Supplementing the joint with lubrication helps decrease the frictional stress exerted on the articular cartilage, thereby effectively maintaining TMJ homeostasis [14]. Hyaluronic acid (HA), a major component of synovial fluid, plays a vital role in lubricating joints, protecting cartilage, and synthesizing proteoglycans within the joint [15]. Previous studies have shown that intra-articular injection of HA can effectively alleviate the signs and symptoms of TMJOA [16,17]. However, rapid clearance and frequent injections for intra-articular HA administration have been associated with complications such as infection, tissue fibrosis, and further joint damage [18]. Moreover, the excessive friction leads to a continuous release of inflammatory factors and catabolic enzymes, which worsens chronic inflammation and disrupts cartilage homeostasis within TMJ [19]. The utilization of microparticles can facilitate the sustained release of therapeutic agents, thereby addressing these drawbacks [10]. Thus, developing HA-based drug delivery microparticles that combine lubrication properties with sustained release of therapeutic agents is expected to be an effective strategy for managing TMJOA.

We aim to employ the excellent lubrication properties of HA and the sustained anti-inflammatory drug release of dynamic boronate ester bonds to prepared HA-based drug-loaded microparticles, validate their protective efficiency of chondrocyte cells under an oxidative stress microenvironment in vitro, and evaluate their therapeutic efficacy for treating TMJOA in vivo.

## Materials and Methods

### Experimental design

**Fig. 1.**
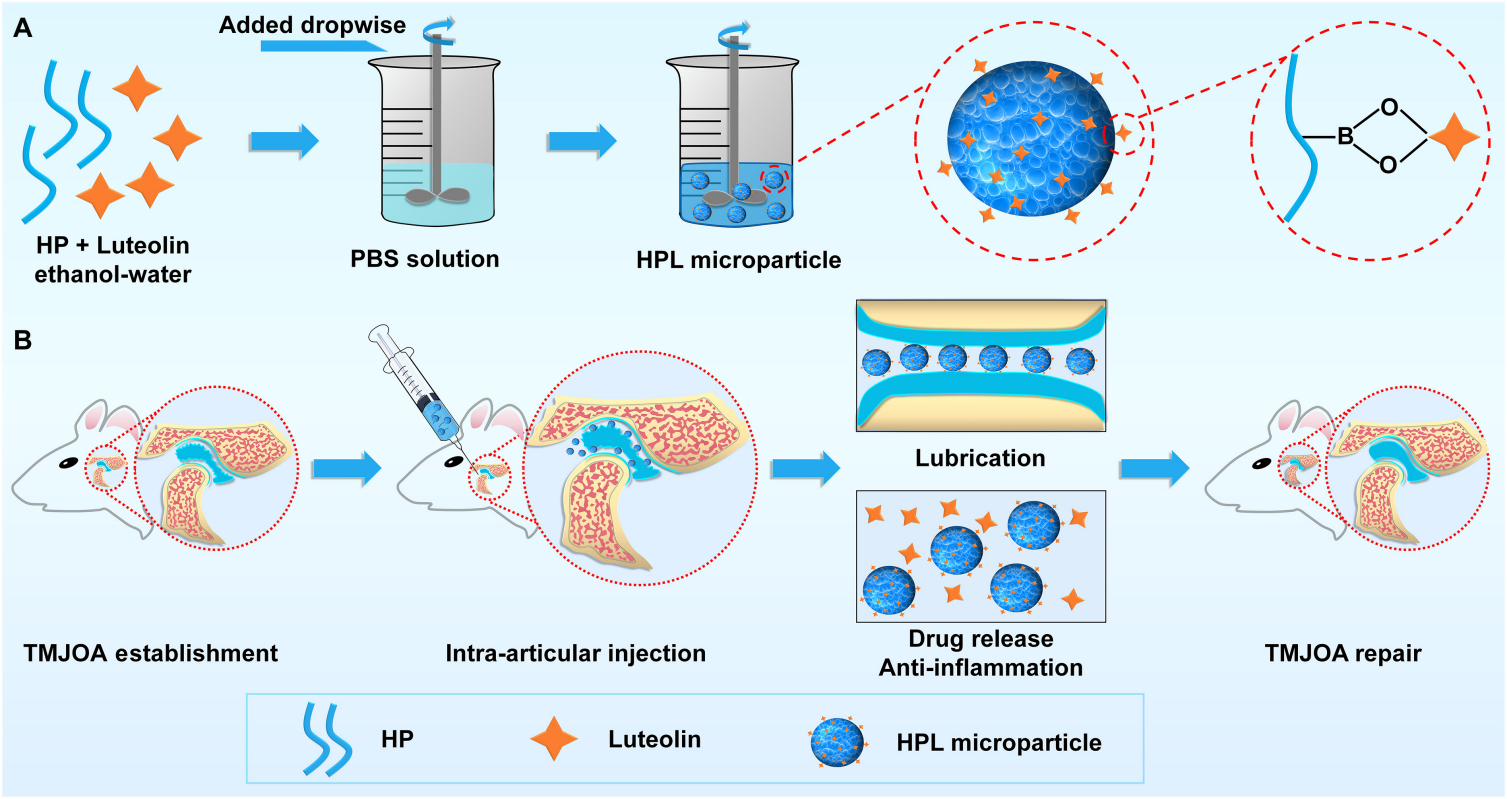
(A) Schematic illustration depicted the fabrication process of luteolin-loaded HP (HPL) microparticles. (B) Treatment of TMJOA through intra-articular injection of HPL microparticles based on the synergistic effect of lubrication, sustained drug release, and anti-inflammatory properties.

In this study, we constructed HA-based microparticles for intra-articular injection, joint lubrication, and sustained drug release in the management of TMJOA (Fig. 1). The 3-aminophenylboronic acid-modified hyaluronic acid (HP) was synthesized and employed for encapsulating luteolin, a common diol-containing anti-inflammatory agent, through dynamic boronate ester bonds. Subsequently, the microparticles were prepared by the self-assembly of HP biopolymer through hydrophobic interaction in an aqueous solution. We systematically characterized the lubrication properties, drug release kinetics, radical scavenging efficiency, and antibacterial activities of the microparticles. Moreover, the cytoprotective effect of drug-loaded microparticles on chondrocyte cells was investigated in vitro under an oxidative stress microenvironment. Finally, the in vivo therapeutic efficacy of drug-loaded microparticles for treating TMJOA was validated through radiological assessment and histological evaluation in a rat model induced by unilateral anterior crossbite (UAC).

### Synthesis of HP

The HP biopolymer was synthesized using the previously reported method with slight modifications [20]. Briefly, 1 g of HA was dissolved in 100 ml of deionized water with vigorous stirring. Then, 0.692 g of 4-(4,6-dimethoxy-1,3,5-triazin-2-yl)-4-methyl morpholinium chloride (DMTMM) and 0.342 g of 3-aminophenylboronic acid (APBA) were added to the HA solution and stirred until they completely dissolved. The pH of the mixture was adjusted to 6.5 by adding 1 M hydrochloric acid. The mixture was allowed to react at room temperature for 72 h, dialyzed with a dialysis tube (7,000 Da, Yuanye, China) for 5 d, and then lyophilized for 2 d to obtain HP.

### Preparation of HP microparticles

HP was first dissolved in a 50% (v/v) ethanol–water solution at 70 °C to achieve a concentration of 10 mg/ml. The 1 ml of aforementioned solution was subsequently added dropwise to 10 ml of phosphate-buffered saline (PBS) solution at a rate of 50 μl/min, with the stirring speed maintained at 1,200 rpm. The resulting mixture was centrifuged to separate the HP microparticles for subsequent analysis.

### Tribological testing of HP microparticles

The lubrication properties of HP microparticles were assessed using an SRV 5 tribometer (Optimal Instruments, Germany) in a linear reciprocating mode. The top surface was composed of a sphere made of polytetrafluoroethylene. The experimental parameters included a frequency of 5 Hz, an oscillation amplitude of 4 mm, an applied load of 10 N, and a test duration lasting for 900 s.

### Radical scavenging efficiency of luteolin-loaded HP microparticles

The HP or HPL microparticles (10 mg) were dispersed in 1 ml of PBS solution and incubated at 37 °C for 12 h. The mixed solution was subsequently centrifuged to obtain the leachate. The radical scavenging assays were conducted using the T-AOC Assay Kit (Beyotime, China) and the 2,2-diphenyl-1-picrylhydrazyl (DPPH) Free Radical Scavenging Capacity Assay Kit (Solarbio, China), following the manufacturers’ instructions.

### Antibacterial activity of HPL microparticles

The antibacterial activity of drug-loaded microparticles was evaluated against *Staphylococcus aureus*. *S. aureus* was cultured in Luria–Bertani broth on a shaker (37 °C, 150 rpm) for 12 h, followed by diluting to a concentration of 10^6^ colony-forming units (CFU)/ml. The HP and HPL microparticle solutions were centrifuged at 12,000 rpm for 5 min. The supernatant was then removed and subsequently redispersed in an equal volume of Luria–Bertani broth. To assess the growth curves of *S. aureus*, the microparticle solutions mentioned above were mixed with bacterial suspension (10^6^ CFU/ml) at a volume ratio of 1:9 and then shaken vigorously for 60 s. The mixed solutions (200 μl) were subsequently transferred to a 96-well plate and incubated in a microplate reader (SPARK, Tecan). The instrument automatically recorded the optical density value at 600 nm every 10 min for a total of 600 min. The growth curves of Con, HP, and HPL groups were drawn based on the optical density values. To determine the bacteriostatic rate, the microparticle solutions mentioned above were mixed with bacterial suspension (10^6^ CFU/ml) at a volume ratio of 1:9 and then shaken vigorously for 60 s. Subsequently, the mixed solutions (200 μl) were evenly spread onto Luria–Bertani agar plates and incubated at 37 °C for an additional 12 h. The bacterial colonies on each plate were counted, and the bacteriostatic rate was determined as follows, where *N* represented the number of bacterial colonies.

$$\begin{array}{c} \text{Bacteriostatic rate}\;\left(\%\right)\;=\;\left(N_{\mathrm{con}}-N_{\text{microparticles}}\right)/N_{\mathrm{con}}\;\times \;100\% \end{array}$$ (1)

### Intracellular reactive oxygen species testing

The intracellular reactive oxygen species (ROS) levels in chondrocyte cells were evaluated using a Reactive Oxygen Species Assay Kit (Beyotime, China) under an oxidative stress microenvironment. Briefly, chondrocyte cells were seeded onto 48-well plates at a density of 10,000 cells per well and cultured for 48 h. The medium was subsequently replaced with a fresh complete medium containing 2 mM H_2_O_2_, followed by the addition of solutions at ^1^/_10_ volume of the complete medium (Con group: PBS solution, HP group: 10 mg of HP microparticles suspended in 1 ml of PBS solution, or HPL group: 10 mg of HPL microparticles suspended in 1 ml of PBS solution). Chondrocyte cells cultured in the normal complete medium were designated as the negative control (NC) group. Chondrocyte cells were cultured for an additional 3 h and then incubated with 2′,7′-dichlorofluorescin diacetate (DCFH-DA) working solution (10 μM) at 37 °C for 20 min. After being rinsed 3 times with a serum-free medium, the chondrocyte cells were observed with a fluorescence microscope (Leica, Germany). The intracellular ROS fluorescence intensity in different groups was quantified using the ImageJ software (National Institutes of Health, USA).

### JC-1 staining

The procedures for cell seeding and culture were the same as mentioned above in the intracellular ROS testing. Chondrocyte cells were cultured for an additional 3 h and then stained with the mitochondrial membrane potential assay kit with 5,5′,6,6′-tetrachloro-1,1′,3,3′-tetraethyl-imidacarbocyanine (JC-1) (Beyotime, China) working solution in accordance with the manufacturer’s instructions. After being rinsed with JC-1 buffer solution, the chondrocyte cells were observed with a fluorescence microscope. The fluorescence intensity of JC-1 aggregates and JC-1 monomers was quantified with the ImageJ software (National Institutes of Health, USA).

### Immunofluorescence staining

The procedures for cell seeding and culture were the same as mentioned above in the intracellular ROS testing. After an additional 6 h of culture, chondrocyte cells were fixed with 4% paraformaldehyde for 10 min, permeated with 0.1% Triton X-100 for 20 min, and then blocked with a solution of 3% bovine serum albumin for 60 min. Chondrocyte cells were incubated overnight with primary antibodies (Aggrecan Polyclonal antibody, 1:400; Collagen Type II Polyclonal antibody, 1:200; Proteintech, USA), stained with Goat Anti-Rabbit IgG H&L (Alexa Fluor 488, 1:600, Abcam, UK) for 1 h, and then stained with Hoechst 33342 (Beyotime, China) for 5 min. After being washed with PBS solutions, the samples were observed with a fluorescence microscope.

### Reverse transcription polymerase chain reaction analysis

To quantify the expression of genes related to anabolism, catabolism, and inflammation, chondrocyte cells were seeded on 6-well plates and cultured until the confluence reached 80% to 90%. The medium was subsequently replaced with fresh complete medium containing 2 mM H_2_O_2_, followed by the addition of solutions of PBS, HP microparticles, or HPL microparticles. After an additional 6 h of culture, the chondrocyte cells were harvested and treated to extract total RNA using a HiPure Total RNA kit (Magentec, China). The extracted total RNA was reverse-transcribed into cDNA using a HiScript III 1st Strand cDNA Synthesis Kit (+gDNA wiper) (Vazyme, China). Subsequently, the expression of anabolism-related genes including collagen type II (COL2) and aggrecan (AGG), catabolism-related genes including a disintegrin and metalloproteinase with thrombospondin motifs 1 (ADAMTS1) and matrix metalloproteinase 13 (MMP13), as well as inflammation-related genes including cyclooxygenase 2 (COX2) and interleukin-1β (IL-1β) was detected using a QuantStudio 5 Flex real-time polymerase chain reaction (PCR) instrument (Life Technologies, USA). β-Actin was used as a reference gene, and the 2^−ΔΔCt^ method was utilized to calculate the relative expression levels of mRNA. The primer sequences were listed in Table S1. The list of acronyms was listed in Table S2.

### Establishment of TMJOA model in rats

All animal experiments were conducted in accordance with the National Research Council’s *Guide for the Care and Use of Laboratory Animals*. All animal procedures were approved by the Animal Ethics and Welfare Committee of the Southern University of Science and Technology (approval number SUSTech-2020-188). Sprague-Dawley rats (6-week-old, female, weighing 200 to 250 g) were obtained from Guangdong Medical Laboratory Animal Center and used for establishing TMJOA model. Nine Sprague-Dawley rats were randomly assigned to 3 experimental groups: Control, HP, and HPL. The TMJOA model was established by UAC induction, as previously reported [21]. Briefly, an 8-mm-long No.20 teat cannula was bent to 135° in angle and 3.5 mm in length. This specialized teat cannula was adhered to the lower left incisor of rats to induce UAC alteration. The metal crowns underwent daily inspections to ensure their proper placement. One week after surgery, samples (50 μl for each sample, Control group: PBS solution, HP group: 10 mg of HP microparticles suspended in 1 ml of PBS solution, or HPL group: 10 mg of HPL microparticles suspended in 1 ml of PBS solution) were injected into the left TMJ cavity of rats through an insulin syringe needle. The intra-articular injection was administered once a week for a total of 4 weeks. After euthanizing the rats, the left TMJ condyles of rats were collected after euthanasia for micro-computed tomography (micro-CT) scanning, histological staining, and immunohistochemical staining.

### Micro-CT scanning

The samples were imaged with a micro-CT system (SkyScan-1276, Bruker) with scanning parameters set as an Al 1-mm filter, a rotation step of 0.4°, and a camera pixel size of 17.450 μm.

### Histological and immunohistochemical staining

After undergoing micro-CT scanning, the samples were fixed in 4% paraformaldehyde solution for 48 h and subjected to a 28-day decalcification process using EDTA decalcified solutions. Then, the samples were embedded in paraffin, sliced into sections with a thickness of 5 μm, and subsequently stained with hematoxylin and eosin (HE) and Safranin-O-Fast green (SF). The destruction of condylar cartilage was assessed using the grading system established by the Osteoarthritis Research Society International (OARSI) [22,23]. For immunohistochemical staining, the sections were incubated overnight at 4 °C with rabbit polyclonal antibodies against COL2, AGG, MMP13, or IL-1β (Servicebio, China). After that, the sections were incubated with goat anti-rabbit secondary antibodies (G1213, Servicebio, China) for 1 h and then treated with 3,3′-diaminobenzidine reagents. The positive staining areas of COL2, AGG, MMP13, and IL-1β were quantified with the ImageJ software (National Institutes of Health, USA).

### Statistical analysis

All data were presented as mean ± standard deviation. Student’s *t* test was employed for comparing 2 groups, and one-way analysis of variance (ANOVA) analysis was used for comparing multiple groups. Statistical analyses were performed by GraphPad Prism 6.0 (GraphPad Software, USA). Statistical significance was defined as follows: *P < 0.05, **P < 0.01, and ***P < 0.001.

## Results

### Synthesis and characterization of HP

**Fig. 2.**
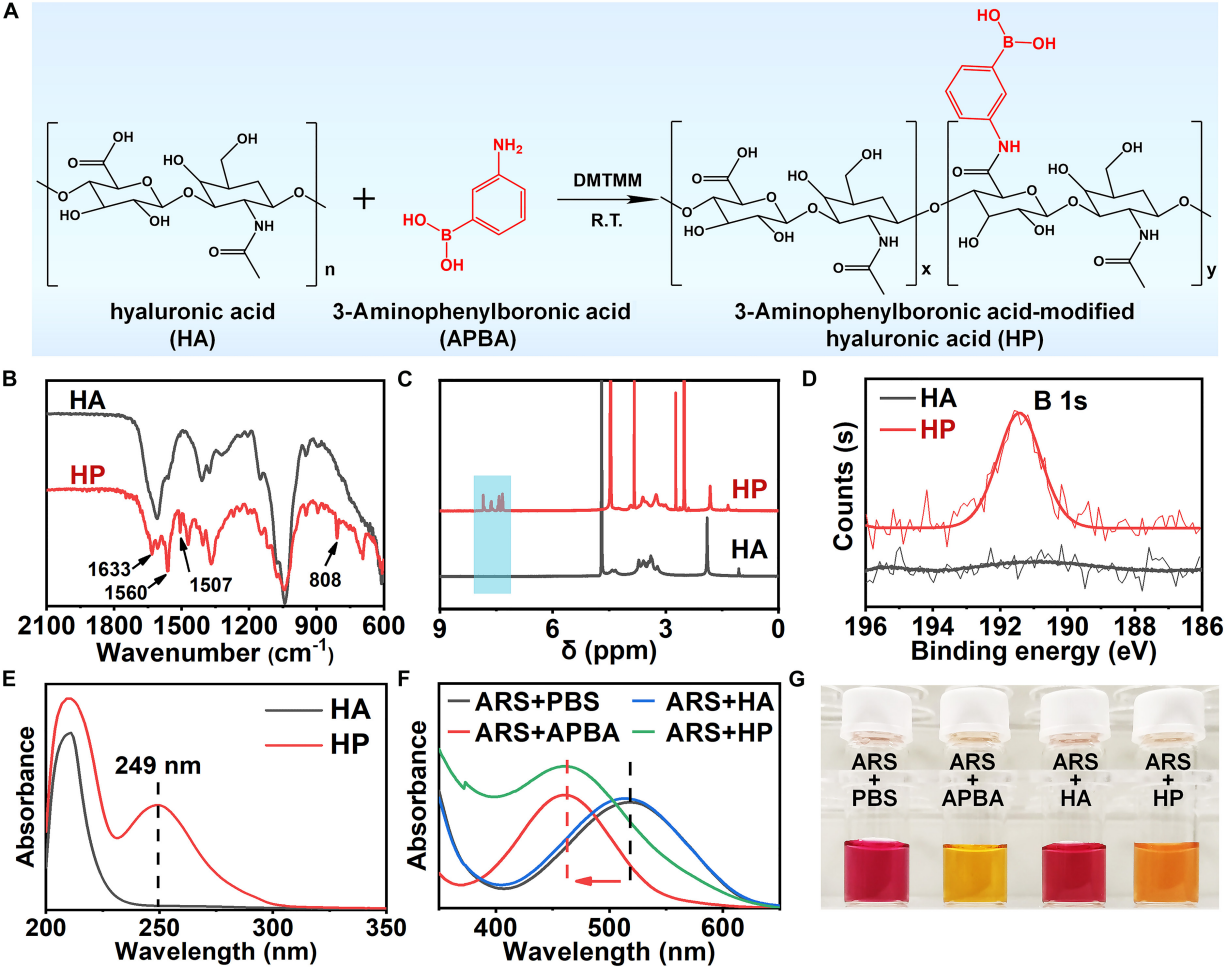
Synthesis and characterization of HP. (A) Schematic illustration of the synthesis process for HP. (B) FTIR spectra, (C) ^1^H NMR spectra, (D) high-resolution XPS spectra of B 1s, and (E) UV spectra of HA and HP. Alteration of (F) UV spectra and (G) color in ARS solution mixed with PBS, APBA, HA, and HP solutions.

Initially, HP was synthesized by chemical modification of HA with APBA through amide linkages using DMTMM as the coupling reagent (Fig. 2A). The chemical structure of HP was characterized by Fourier transform infrared (FTIR) spectroscopy, ^1^H nuclear magnetic resonance (NMR) spectra, x-ray photoelectron spectroscopy (XPS), and ultraviolet-visible (UV-vis) spectroscopy. As shown in Fig. 2B, when compared to HA, the FTIR spectrum of HP exhibited noticeable characteristic peaks at 1,633 cm^−1^ and 1,560 cm^−1^, which corresponded to the amide I and amide II bands, respectively. HP also showed a characteristic respiration vibration of the benzene ring at 1,507 cm^−1^ and an out-of-plane bending vibration peak of meta-disubstituted C–H at 808 cm^−1^. Moreover, the ^1^H NMR spectrum of HP revealed the emergence of new peaks at 7.2 to 7.9 ppm, which were attributed to the phenylboronic acid groups (Fig. 2C). The B 1s signal at 191.4 eV and a maximal absorbance peak at 249 nm were also detected in the XPS and UV-vis spectra of HP, respectively (Fig. 2D and E and Fig. S1). Gel permeation chromatography (GPC) analysis in Fig. S2 showed that HP exhibited a decrease in molecular weight (*M*_n_ = 70 kg/mol) and an increase in polydispersity index (*M*_w_/*M*_n_ = 2.78) compared to HA (*M*_n_ = 516 kg/mol and *M*_w_/*M*_n_ = 1.61). Furthermore, we established the standard curve of APBA (Fig. S3) and determined that the APBA content in HP materials was 1.45 ± 0.06 mmol/g.

Alizarin red S (ARS) complexation assay was conducted to confirm the conjugation between diol-containing molecules and boronic acid groups in HP backbones [20]. The color of ARS solution changed from deep red to yellow and orange-yellow upon addition of APBA and HP solution, respectively (Fig. 2G). This phenomenon was accompanied by a distinct shift in the maximal absorbance peak from 520 nm to 460 nm, indicating the formation of ARS complex (Fig. 2F). It should be mentioned that the content of APBA in dialysis water was also monitored during purification using the ARS complexation assay. The results showed that the unreacted APBA could be effectively removed from the reaction solution through dialysis (Fig. S4). Taken together, these results demonstrated the successful grafting of APBA onto HA, and the resultant HP retained the capacity to form boronate ester bonds with diol-containing molecules.

### Preparation and characterization of HP microparticles

**Fig. 3.**
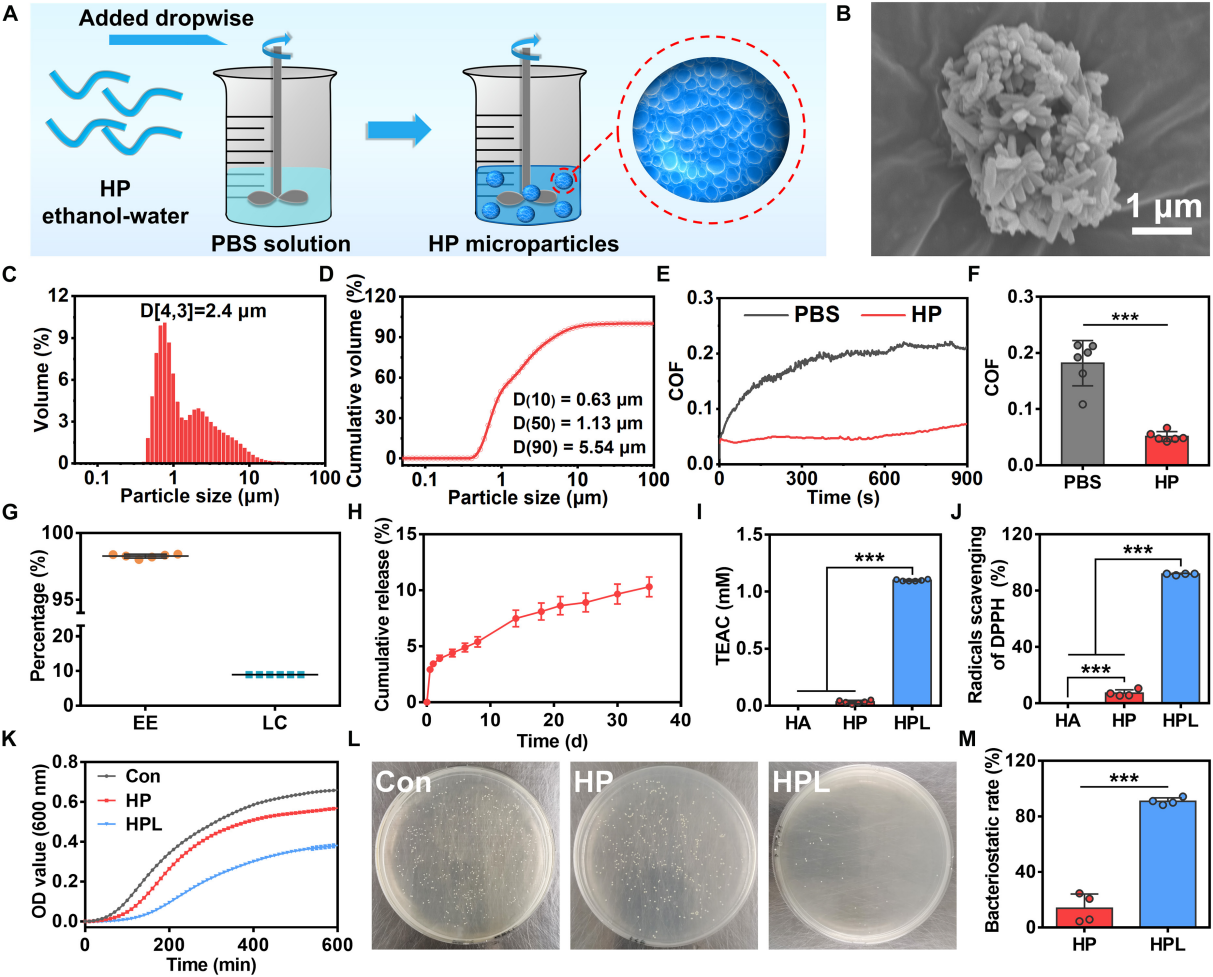
Preparation and characterization of the HP microparticles. (A) Schematic illustration depicted the fabrication process of HP microparticles. (B) SEM images, (C) particle size distribution, and (D) cumulative distribution of HP microparticles. (E) COF–time curves and (F) COF values of PBS and HP microparticle solutions. (G) Drug loading and (H) cumulative release curve of HPL microparticles in PBS solution (*n* = 6). (I) TEAC of HP and HPL microparticles against ABTS radicals (*n* = 6). (J) DPPH radicals scavenging of the HP and HPL microparticles (*n* = 4). (K) Growth curves, (L) bacterial colonies on Luria–Bertani agar plates, and (M) bacteriostatic rate of *S. aureus* cultured with HP and HPL microparticles (*n* = 4). Statistical analysis was conducted using Student’s *t* test between 2 groups and one-way ANOVA analysis among multiple groups. Data were represented as mean ± standard deviation. *P < 0.05, **P < 0.01, ***P < 0.001.

HP exhibited significantly lower water solubility compared to HA, which provided a straightforward method for preparing microparticles in aqueous solutions (Fig. S5). As shown in Fig. 3A, the HP microparticles were obtained by adding an ethanol–water solution of HP dropwise into an excess aqueous solution using a microfluidic device. The formation of microparticles can be attributed to the self-assembly of HP biopolymer through hydrophobic interactions in an aqueous solution. The scanning electron microscope (SEM) image revealed microparticles exhibiting typical aggregate structures (Fig. 3B). Next, we employed the laser diffraction technique to measure the particle size distribution of HP microparticles. The volume moment mean, also known as the De Brouckere mean diameter (D[4,3]), is a commonly used parameter that accurately represents the particle size [24]. The D[4,3] of HP microparticles was determined to be approximately 2.4 μm (Fig. 3C). Moreover, Fig. 3D displayed the diameters of microparticles at cumulative volumes of 10%, 50%, and 90%, which was denoted as D (10), D (50), and D (90), respectively. The D (90) of the microparticles was less than 5.5 μm, allowing for easy injection through an insulin syringe needle (Fig. S6).

Previous studies have demonstrated that the injection of super-lubricating microparticles can effectively enhance lubrication of the cartilage surface and reduce mechanical friction-induced damage to articular cartilage [25,26]. We then measured the coefficient of friction (COF) value of the HP microparticle solution using a tribometer. The HP microparticles exhibited a smoother friction coefficient curve and a significantly lower COF values compared to PBS solution (Fig. 3E and F). Furthermore, although the COF values of HP microparticles increased after 4 repetitive cycles, they remained lower than that of PBS solution (Fig. S7), indicating stable and superior lubrication properties of the HP microparticles.

### Drug loading and release of HPL microparticles

The HPL microparticles were prepared using a similar method as the HP microparticles. To evaluate the encapsulation efficiency, loading capacity, and release kinetics of luteolin from the HPL microparticles, a standard curve was first established by plotting the UV absorbance of luteolin at 348 nm against its concentration (Fig. S8). The HP microparticles could effectively load luteolin with an encapsulation efficiency of 98.3 ± 0.1% and a loading capacity of 8.93 ± 0.01% (Fig. 3G). Additionally, the conjunction of luteolin and HP through boronate ester bonds resulted in a decrease in drug release rate [27]. Approximately 10% of luteolin was released under physiological conditions on day 35, demonstrating the sustained drug release behavior of HPL microparticles (Fig. 3H). This property is particularly beneficial for reducing the number of injections required for TMJOA treatment, thereby relieving patients’ suffering.

### Antioxidant and antibacterial activities

The patients with TMJ disorder demonstrate overexpressed oxidative stress markers and diminished total antioxidant capacity, indicating the therapeutic potential of interventions with antioxidant activity and radical scavenging efficiency [10]. Thus, the antioxidant capacity of HPL microparticles was assessed by measuring the radical scavenging efficiency towards both 2,2′-azino-bis (3-ethylbenzthiazoline-6-sulfonic acid (ABTS) and DPPH free radicals. Trolox equivalent antioxidant capacity (TEAC) assay was conducted to evaluate the ABTS radical scavenging ability, with a higher TEAC value indicating greater scavenging activity. Notably, the HPL group exhibited a significantly higher TEAC value than both the HA and HP groups (Fig. 3I). Moreover, the ability of HPL to scavenge DPPH free radicals was assessed by monitoring the absorbance at 515 nm. The HPL group exhibited significantly higher efficiency in scavenging DPPH radicals (over 90%) compared to the HA and HP groups (Fig. 3J). The remarkable antioxidant activity of HPL could be attributed to the presence of reductive phenolic hydroxyl groups in luteolin, which showed promising potential for the treatment of OA.

An optimal intra-articular drug delivery system should possess certain antibacterial activity against *S. aureus*, which is the most prevalent pathogen found in bone and joint infections [28,29]. We assessed the potential antibacterial activities of HPL microparticles. The bacteria growth curves in Fig. 3K showed that the HPL microparticles effectively inhibited the growth of *S. aureus* compared with the HP microparticles. Moreover, the HPL group exhibited a bacteriostatic rate of 84%, which was significantly higher than the HP group’s rate of 15% (Fig. 3L and M), indicating the potential of HPL microparticles in preventing joint infection caused by *S. aureus*.

### In vitro cytoprotective effect of HPL microparticles on chondrocyte cells within oxidative stress microenvironment

Biocompatibility is a fundamental prerequisite for clinical applications [30,31]. The cell viability of HP and HPL microparticles remained above 85% after incubation with chondrocyte cells in normal complete medium, indicating that the microparticles were not cytotoxic (Fig. S9). H_2_O_2_ is commonly employed as a potent oxidizing agent to simulate the oxidative stress microenvironment associated with OA [32]. Therefore, we established an in vitro oxidative stress microenvironment by utilizing various concentrations of H_2_O_2_ to mimic the different levels of ROS present in TMJOA. As shown in Fig. S10, the activity of chondrocyte cells decreased gradually with the increasing concentrations of H_2_O_2_. Moreover, Alcian blue and Safranin-O staining were conducted to assess the secretion of extracellular matrix (ECM). The chondrocyte cells exhibited cellular aggregation and ECM atrophy when the concentration of H_2_O_2_ reached or exceeded 2 mM (Fig. S11). Thus, a concentration of 2 mM H_2_O_2_ was chosen as appropriate for the subsequent cytoprotective experiments in vitro.

**Fig. 4.**
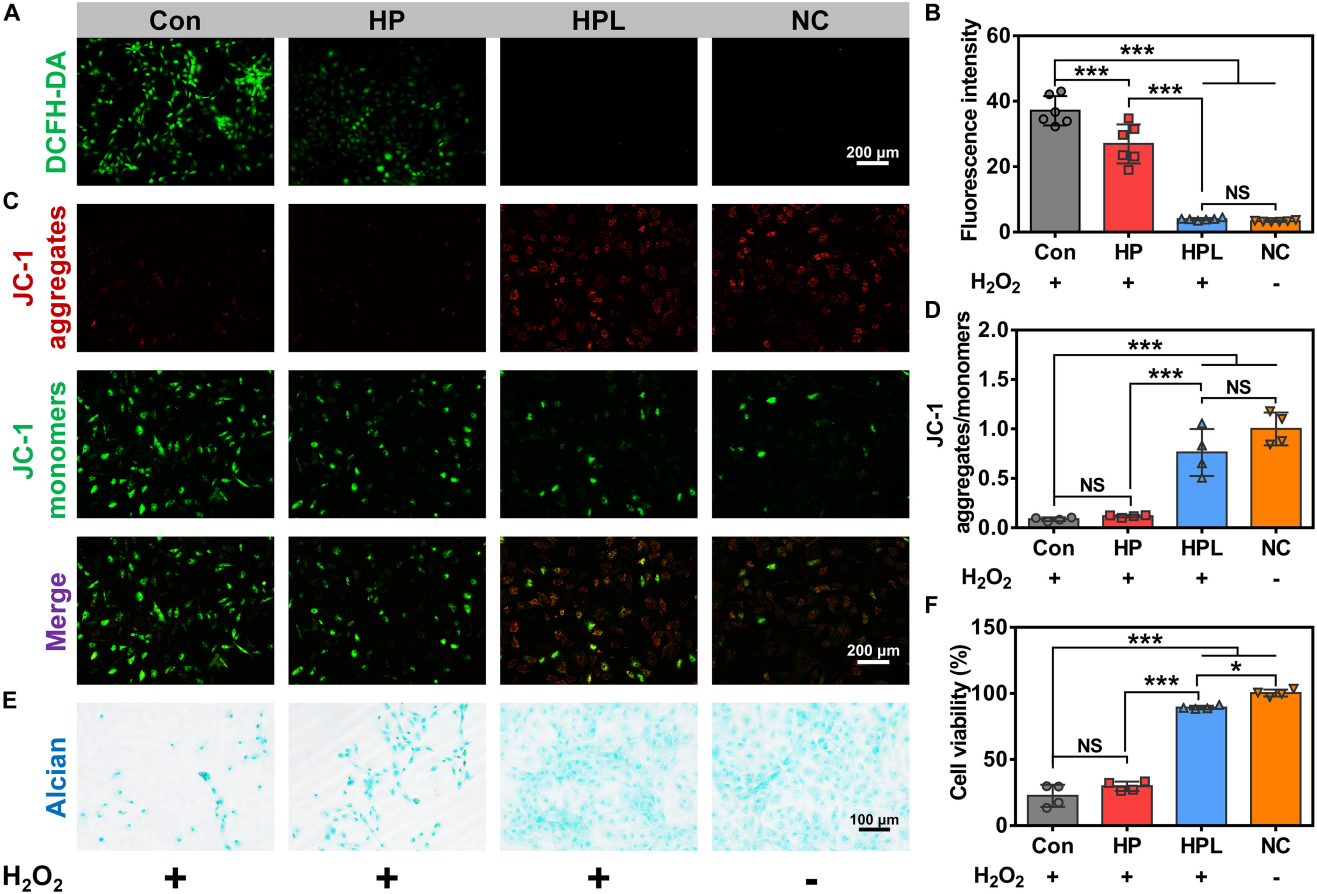
Cytoprotective effect of HPL microparticles on chondrocyte cells under oxidative stress microenvironment. (A) Representative DCFH-DA staining images, (B) fluorescence intensity statistics of DCFH-DA staining (*n* = 6), (C) representative JC-1 staining images, and (D) fluorescence intensity statistics of JC-1 staining (*n* = 4) of chondrocyte cells cultured with HP and HPL microparticles for 3 h under oxidative stress microenvironment. (E) Representative Alcian blue staining images and (F) CCK-8 assay (*n* = 4) of chondrocyte cells cultured with HP and HPL microparticles for 6 h under oxidative stress microenvironment. Statistical analysis was conducted using one-way ANOVA analysis. Data were represented as mean ± standard deviation. *P < 0.05, **P < 0.01, ***P < 0.001, NS: not significant.

We further conducted a comprehensive cytological analysis, including intracellular ROS levels, mitochondrial membrane potential, ECM secretions, and expression levels of genes and proteins related to ECM balance, to evaluate the potential cytoprotective effect of HPL microparticles on chondrocyte cells under oxidative stress microenvironment. Chondrocyte cells cultured in a normal complete medium were designated as the NC group, while those cultured with the addition of H_2_O_2_ were assigned to the Con group. The intracellular ROS levels were detected with a DCFH-DA probe, which indicated higher intracellular ROS levels with stronger green fluorescence [33]. As shown in Fig. 4A, the chondrocyte cells cultured in the presence of HPL microparticles exhibited a significantly lower fluorescence level compared to those in the Con and HP groups under the oxidative stress microenvironment. Notably, comparable fluorescence signals were found between the HPL and NC groups. Further quantitative analysis revealed that the fluorescence intensity for the HPL group was approximately 11% and 15% of that in the Con and HP groups, respectively. No significant difference was observed in the HPL and NC groups (Fig. 4B), which was consistent with the results of fluorescence images. The results demonstrated that the HPL microparticles effectively mitigated the intracellular ROS induced by H_2_O_2_.

JC-1 has been widely employed for the rapid detection of changes in mitochondrial membrane potential to identify early apoptosis of cells [34]. As shown in Fig. 4C, the normal chondrocyte cells (NC group) exhibited robust staining of JC-1 aggregates (red fluorescence) and weak staining of JC-1 monomers (green fluorescence) after being treated with the JC-1 probe. When exposed to H_2_O_2_, a significant increase in JC-1 monomers (green fluorescence) and a remarkable decrease in JC-1 aggregates (red fluorescence) were observed in the Con and HP groups, indicating severe mitochondrial dysfunction and the onset of early apoptosis. In sharp contrast, the HPL group exhibited a similar fluorescence intensity for both JC-1 aggregates and monomers as the NC group. Furthermore, the quantitative analysis confirmed that there was no statistical difference in the JC-1 aggregate/monomer ratio between the HPL group and the NC group, but it was significantly higher than those of the Con and HP groups (Fig. 4D and Fig. S12). These results highlighted the inhibitory effect of HPL microparticles on early apoptosis under oxidative stress microenvironment.

After subjecting chondrocyte cells to prolonged exposure in an oxidative stress microenvironment, we further validated the cytoprotective capability of HPL microparticles. The HPL group showed comparable ECM secretion to the NC group, while the Con and HP groups exhibited significantly lower ECM secretion compared with the HPL and NC groups (Fig. 4E). Moreover, the cell viability in the HPL group was 89.3% of that in the NC group, which was significantly higher than 22.6% and 29.8% in the Con and HP groups, respectively (Fig. 4F). Overall, these results suggested a favorable cytoprotective effect of HPL microparticles on chondrocyte cells under oxidative stress microenvironment in vitro, which was believed to be attributed to its excellent antioxidant activity.

**Fig. 5.**
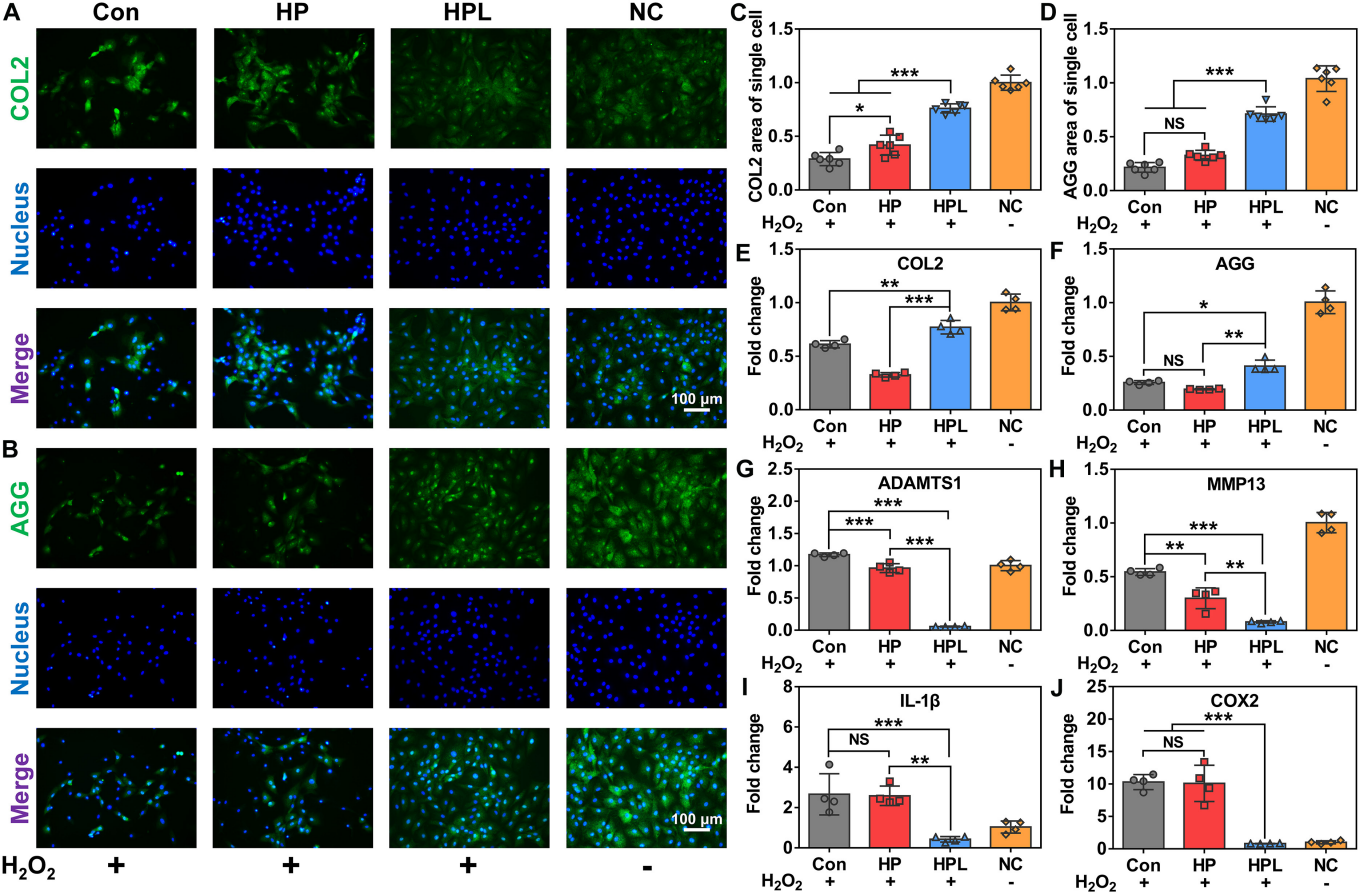
Cytoprotective effect of HPL microparticles on chondrocyte cells under oxidative stress microenvironment. (A) Representative COL2 staining images, (B) AGG staining images, (C) fluorescence intensity statistics of COL2 staining, and (D) fluorescence intensity statistics of AGG staining of chondrocyte cells cultured with HP and HPL microparticles for 6 h under oxidative stress microenvironment (*n* = 6). The gene expression levels of (E) COL2, (F) AGG, (G) ADAMTS1, (H) MMP13, (I) IL-1β, and (J) COX2 of chondrocyte cells cultured with HP and HPL microparticles for 6 h under oxidative stress microenvironment (*n* = 4). Statistical analysis was conducted using one-way ANOVA analysis. Data were represented as mean ± standard deviation. *P < 0.05, **P < 0.01, ***P < 0.001, NS: not significant.

Next, the capacity of HPL microparticles to modulate the balance of ECM was evaluated at both the gene and protein levels. The robust synthesis of COL2 and AGG is a crucial feature of healthy chondrocyte cells [35]. The results of immunofluorescence staining revealed significantly higher expression levels of COL2 (Fig. 5A and 5C and Fig. S13A) and AGG (Fig. 5B and D and Fig. S13B) in the HPL group compared to those observed in the Con and HP groups. Furthermore, the expression levels of anabolism-related genes (COL2 and AGG) were significantly up-regulated in the HPL group compared with the Con and HP groups (Fig. 5E and F). Meanwhile, the expression levels of catabolism-related genes, including ADAMTS1 and MMP13, were significantly down-regulated in the HPL group compared to those in the Con and HP groups (Fig. 5G and H). The results of gene and protein expression analysis suggested that the HPL microparticles effectively inhibited ECM degradation and promoted ECM regeneration. In addition, the presence of inflammatory cytokines, such as IL-1β and COX2, has been reported to accelerate ECM matrix degradation, enhance matrix metalloproteinase production, and promote chondrocyte cell apoptosis [36,37]. As shown in Fig. 5I and J, the HPL group exhibited a significant down-regulation of inflammation-related genes (IL-1β and COX2) compared to the Con and HP groups, indicating that HPL microparticles effectively inhibited the inflammatory response of chondrocyte cells under oxidative stress microenvironment. Taken together, these results confirmed the efficacy of HPL microparticles in restoring the balance between ECM synthesis and ECM catabolism, as well as inhibiting the inflammatory response.

### In vivo treatment of TMJOA by intra-articular injection of HPL microparticles

**Fig. 6.**
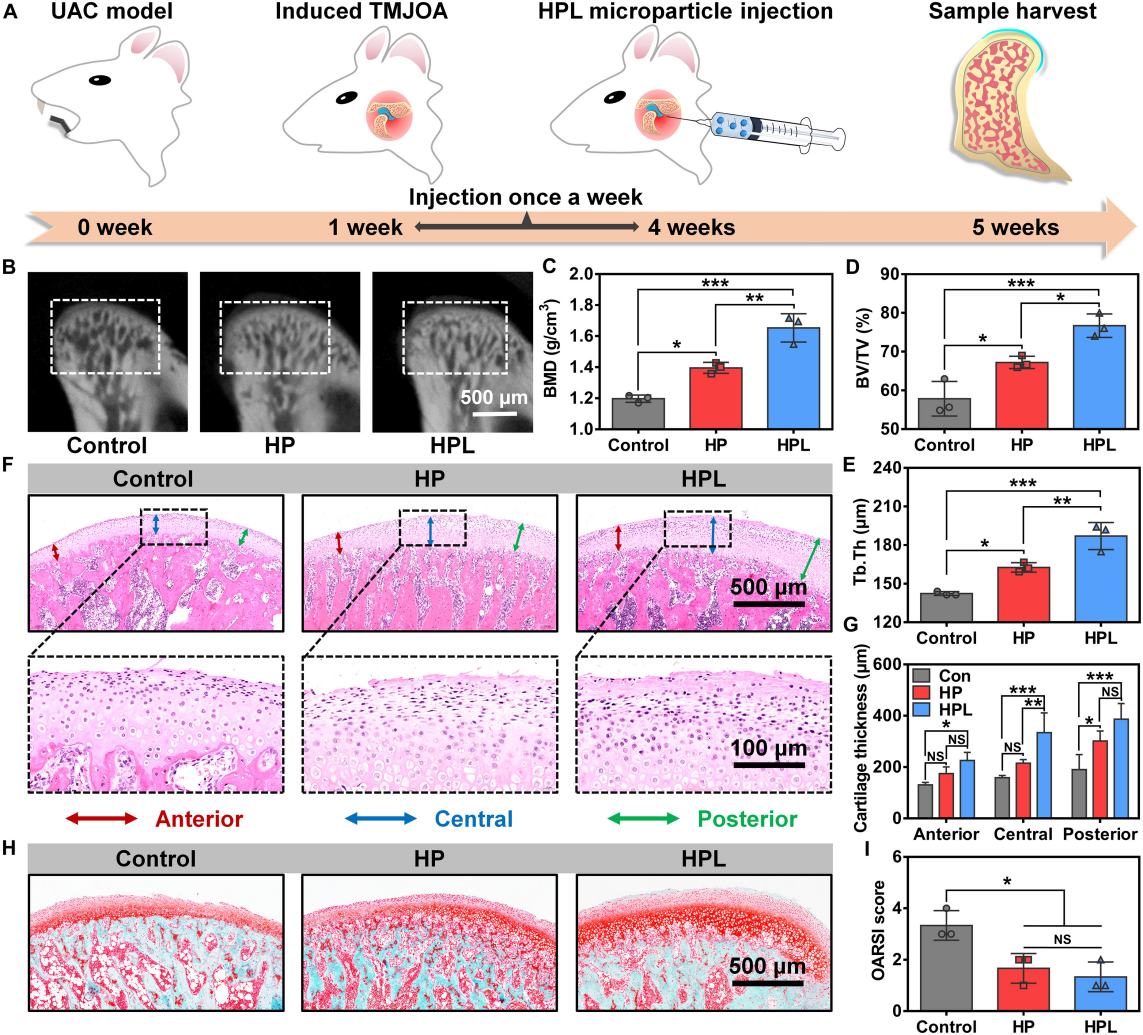
In vivo therapeutic effect of HPL microparticles on the treatment of TMJOA in a rat model. (A) Schematic illustration of TMJOA induced by the UAC model and the treatment process through intra-articular injection of HPL microparticles. (B) Representative sagittal images as well as statistical analysis of (C) bone mineral density (BMD), (D) bone volume to tissue volume ratio (BV/TV), and (E) trabecular thickness (Tb.Th) based on micro-CT analysis at week 5 after surgery. (F) HE staining of rat condylar cartilage sections for the Control, HP, and HPL groups. (G) Cartilage thickness was evaluated from the HE staining in the testing directions of anterior, central, and posterior. (H) SF staining of rat condylar cartilage sections for the Control, HP, and HPL groups. (I) OARSI score of the condylar samples in the Control, HP, and HPL groups. Statistical analysis was conducted using one-way ANOVA analysis. Data were represented as mean ± standard deviation. *n* = 3 individual rats. *P < 0.05, **P < 0.01, ***P < 0.001, NS: not significant.

The in vivo therapeutic efficacy of HPL microparticles in treating TMJOA was further investigated in the rat model, which was induced using a widely employed UAC model [13,38]. A crossbite-leading crown procedure was performed on the rats, followed by weekly injections of HPL microparticles into the TMJ capsules for 4 doses (Fig. 6A). The mandibular condylar samples were harvested for micro-CT, histological, and immunohistochemical analyses at week 5 after surgery. Representative micro-CT images in Fig. 6B illustrated that distinct hollow regions were observed within the subchondral bone in the Control group, while intra-articular injection of microparticles (HP and HPL) resulted in significantly reduced hollow areas compared with the Control group, particularly in the HPL-treated rats. To quantitatively evaluate the bone quality of each sample, we characterized histomorphometric parameters including bone mineral density (BMD), bone volume fraction (BV/TV), trabecular thickness (Tb.Th), trabecular number (Tb.N), and trabecular separation (Tb.Sp) based on micro-CT images. Notably, the HPL group exhibited significantly higher BMD, BV/TV, Tb.Th, and Tb.N compared to both the HP and Control groups while demonstrating a considerably lower Tb.Sp (Fig. 6C to E and Fig. S14). These results suggested that the intra-articular injection of HPL microparticles could effectively mitigate osteoporosis-like bone damage.

The condylar cartilage was further observed using HE and SF staining at week 5 after surgery. The results from HE staining demonstrated evidence of cartilage abrasion and disrupted cartilage structure in the Control group, indicating the presence of OA (Fig. 6F). In contrast, the rats treated with HPL microparticles maintained smooth cartilage surfaces and exhibited typical hierarchical structures that were present in healthy cartilage [39]. Moreover, the HPL group also showed a significantly greater cartilage thickness compared to the Control group, regardless of the testing directions (anterior, central, and posterior) (Fig. 6G). The SF staining in Fig. 6H also revealed intact cartilage with evenly arranged cells in each layer for microparticle-treated rats, especially in the HPL group. This was in sharp contrast to the Control group, where cartilage thinning and irregularly arranged cartilage were observed. The OARSI grade is based on pathological sections of articular cartilage, where a lower score indicates a more favorable condition of the cartilage [22]. As shown in Fig. 6I, the microparticle-treated groups demonstrated significantly lower scores compared to those of the Control group. Overall, these results demonstrated the effective alleviation of TMJOA through the intra-articular injection of HPL microparticles, as evidenced by the restored condylar structure, improved cartilage thickness, and facilitated matrix deposition.

**Fig. 7.**
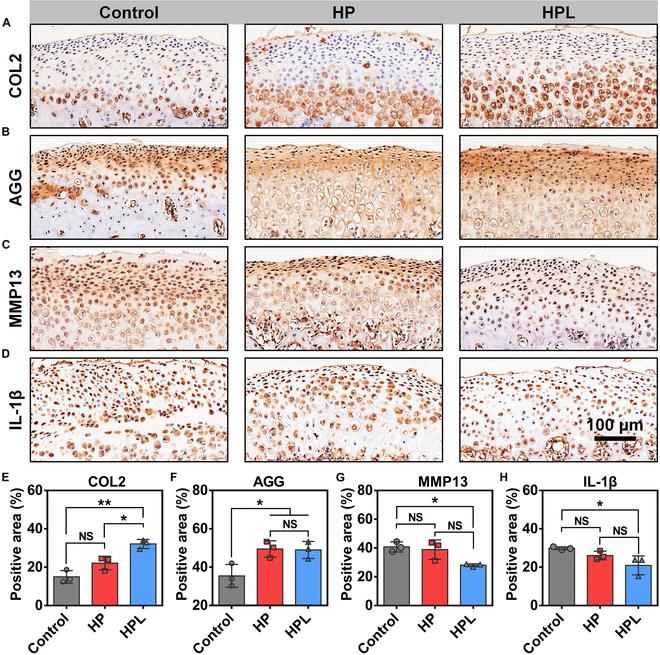
Immunohistochemical analysis of COL2, AGG, MMP13, and IL-1β expressions of rat condylar cartilage sections at week 5 after surgery. Representative immunohistochemical staining images of (A) COL2, (B) AGG, (C) MMP13, and (D) IL-1β. The quantitative analysis of (E) COL2, (F) AGG, (G) MMP13, and (H) IL-1β-positive staining areas. Statistical analysis was conducted using one-way ANOVA analysis. Data were represented as mean ± standard deviation. *n* = 3 individual rats. *P < 0.05, **P < 0.01, ***P < 0.001, NS: not significant.

The condylar cartilage sections were further subjected to immunohistochemical staining for anabolism-related markers (COL2 and AGG), catabolism-related marker (MMP13), as well as inflammation-related marker (IL-1β) analyses. As shown in Fig. 7A to C, weaker staining of COL2 and AGG, as well as pronounced staining of MMP13 were observed in the Control group, indicating the occurrence of obvious cartilage degradation. However, the microparticle-treated groups exhibited an inhibition of cartilage degradation in the UAC model, as demonstrated by increased positive staining areas for COL2 and AGG, along with decreased positive staining areas for MMP13. Additionally, there were fewer positive staining areas of IL-1β in the microparticle-treated groups compared with the Control group, demonstrating a lower degree of inflammatory response in the microparticle-treated groups (Fig. 7D). Quantitative analysis of the positive staining areas was summarized in Fig. 7E to H. The HPL group exhibited significantly higher positive staining areas of COL2 and AGG, as well as remarkably lower positive staining areas of MMP13 and IL-1β compared to the Control group. These findings indicated that the intra-articular injection of HPL microparticles could effectively alleviate TMJOA by promoting matrix regeneration and suppressing inflammatory response. Overall, we confirmed the in vivo efficacy of HPL microparticles in mitigating cartilage damage and alleviating the progression of TMJOA.

## Discussion

TMJOA is widely recognized as a typical disease strongly associated with friction [9]. Excessive mechanical loading can initiate cartilage wear, exacerbate the inflammatory response, and accelerate the progression of TMJOA [13]. Therefore, reducing friction and inflammation is essential for the treatment of TMJOA. Clinically, the most commonly utilized agents include HA, which supplements viscosity, as well as corticosteroids and nonsteroidal anti-inflammatory drugs (NSAIDs), known for their anti-inflammatory efficacy [40]. Nevertheless, the rapid clearance and frequent administration of synovial fluid or anti-inflammatory agents have been associated with complications such as infection, tissue fibrosis, and further joint damage [18]. To address these issues, we developed injectable drug-loaded microparticles for intra-articular treatment of TMJOA to provide lubrication and facilitate long-term anti-inflammatory effects synergistically.

Supplementing lubricating fluid, such as HA solution, helps to reduce frictional stress and maintain TMJ homeostasis [14], as reported in the previous studies [2,16,19]. Compared to lubricating fluid, nanoparticles/microparticles can also be evenly distributed on the joint surfaces, forming lubricating layers that significantly reduce interfacial friction between joint surfaces [15]. Some nanoparticles/microparticles can create a rolling effect between the contact surfaces, known as rolling lubrication, which effectively reduces COF [41]. More important, these nanoparticles/microparticles offer multifunctional characteristics such as drug-loading capabilities, targeted delivery, and intelligent response [42]. Thus, we have chosen HA as the fundamental material, successfully modified HA with phenylboronic acid groups for drug loading (Fig. 2), and prepared it into microparticles (Fig. 3A to D). Boronic acids have a high tendency to form dynamic covalent boronate esters with 1,2- and 1,3-diols, which are responsible for inducing self-healing ability in the materials [43]. This means that phenylboronic acid-modified HA can spontaneously load drugs containing 1,2- and 1,3-diols. Furthermore, the inevitable rearrangement of boronate esters, including hydrolysis to yield the free boronic acids and diols, as well as the reformation of boronate ester, enables HP microparticles and diol-containing drugs to exhibit a “self-healing” behavior similar to that of boronate ester hydrogels [44]. The prepared microparticles maintained injectability and demonstrated a low COF value (Fig. 3E and F), suggesting their potential application in TMJOA.

Excessive friction leads to the continuous release of inflammatory factors and catabolic enzymes, which worsens chronic inflammation and disrupts cartilage homeostasis within TMJ [19]. Therefore, appropriate anti-inflammatory agents are crucial for treating TMJOA. Luteolin, an important natural flavonoid, is widely distributed throughout the plant kingdom and contains diol structures [45]. It has been reported that luteolin possesses a diverse range of pharmacological activities, including anti-inflammatory property, antioxidant activity, antitumor efficacy, neuroprotective effect, and the ability to inhibit various bacteria and viruses [46,47]. Therefore, luteolin was selected as a model drug and successfully encapsulated in HP microparticles, which exhibited sustained drug release (Fig. 3G and H). *S. aureus* is the most prevalent pathogen found in bone and joint infections [28,29]. The HPL group exhibited a certain antibacterial activity, indicating its potential in preventing joint infection caused by *S. aureus* (Fig. 3K to M).

Mechanical stress or inflammatory reactions in joint tissues increase the levels of ROS, leading to cartilage destruction in TMJ [48]. The patients with TMJ disorder demonstrate overexpressed oxidative stress markers and diminished total antioxidant capacity [10]. Therefore, it is necessary to control the production of ROS during inflammation. HPL exhibited excellent antioxidant properties (Fig. 3I and J) and remarkable cytoprotective effects on chondrocyte cells under an oxidative stress microenvironment in vitro (Figs. 4 and 5). Additionally, the HPL microparticles demonstrated therapeutic effect in mitigating cartilage damage and alleviating the progression of TMJOA in vivo (Figs. 6 and 7). The synergistical treatment results of lubrication and anti-inflammation were consistent with other reports [19,21].

Despite the exciting results of this study, there are still several limitations that need to be addressed. The animal experiments exclusively focused on female rats, necessitating the consideration of both gender and species in future studies. Furthermore, the preparation method should be further improved to include nanoparticles in the scope of research. Moreover, the uptake of these microparticles by cells is limited; however, the fragmented particles caused by repetitive pressure may enter the intracellular space. Therefore, more in-depth research should be conducted on the impact of fragmented particles on therapeutic effects.

In summary, we have reported a drug-loaded microparticle based on modified HA biopolymer with enhanced lubrication properties, antioxidant activities, and anti-inflammatory effects for the treatment of TMJOA. The microparticles self-assembled through hydrophobic interactions of HP in an aqueous solution, and a diol-containing anti-inflammatory agent was encapsulated within the microparticles through dynamic boronate ester bonds. The resulting microparticles showed excellent injectability, lubrication properties, radical scavenging efficiency, and antibacterial activities. Additionally, the drug-loaded microparticles exhibited a favorable cytoprotective effect on chondrocyte cells in vitro under the oxidative stress microenvironment. In vivo experiments demonstrated that intra-articular injection of drug-loaded microparticles effectively alleviated osteoporosis-like bone damage, suppressed inflammatory response, and facilitated matrix regeneration in the treatment of TMJOA. The HP microparticle exhibited excellent injectability and encapsulation capacity for diol-containing drugs, highlighting its potential as a versatile drug delivery vehicle in the intra-articular treatment of TMJOA.

## Data Availability

Data will be made available on reasonable request.

## Supplementary Materials

Supplementary 1 Materials and Methods Notes S1 to S9 Figs. S1 to S14 Tables S1 and S2 Movies S1 to S4 References
