# Supplementary material for: Hyaluronic Acid-Based Microparticles with Lubrication and Anti-Inflammation for Alleviating Temporomandibular Joint Osteoarthritis
Source: Biomater Res. 2024 Sep 6;28:0073. doi: 10.34133/bmr.0073 (PMC11377958; doi:10.34133/bmr.0073)
Supplement: Supplementary 1 — Materials and Methods Notes S1 to S9 Figs. S1 to S14 Tables S1 and S2 Movies S1 to S4 References [file bmr.0073.f1.pdf]

Supplementary Materials

Hyaluronic Acid-Based Microparticles with Lubrication and Anti-inflammation for Alleviating Temporomandibular Joint Osteoarthritis

Lei Liu[1†], Gang He[1†], Yixi Li[1], Yiwen Xian[1], Guixian He[2], Yonglong Hong[2*], Chong Zhang[1*], and Decheng Wu[1*]

[1]Guangdong Provincial Key Laboratory of Advanced Biomaterials, Department of Biomedical Engineering, Southern University of Science and Technology, Shenzhen 518055, China. [2]Department of Maxillofacial Surgery, Shenzhen Hospital, Southern Medical University, Shenzhen 518101, China.

[*]Address correspondence to: wudc@sustech.edu.cn (D. W.); zhangc9@sustech.edu.cn (C. Z.); ylhong93@163.com (Y. H.)
†These authors contributed equally to this work.

Materials and methods

Materials

Hyaluronate sodium (molecular weight 1-1.5 million Da) was purchased from Shanghai yuanye Bio-Technology Co., Ltd. (China). Luteolin and 3-aminophenylboronic acid (APBA) were obtained from Energy Chemical (China). 4-(4,6-Dimethoxy-1,3,5-triazin-2-yl)-4-methyl morpholinium chloride (DMTMM) and Alizarin red S (ARS) were purchased from Aladdin (China). Fetal bovine serum was obtained from ExCell Bio. (China). Dulbecco's modified eagle medium (DMEM) and penicillin-streptomycin were purchased from Gibco (USA).

Characterization of HP

The Fourier transform infrared (FTIR) spectra of HA and HP were measured using an iS20 FTIR Spectrometer (Thermo Fisher Scientific, USA) equipped with an attenuated total reflectance module. The proton nuclear magnetic resonance (^{1}H NMR) spectra were obtained using an Avance NEO 600 spectrometer (Bruker, Germany). The HA was dissolved in deuterated water at a concentration of 10 mg/mL, and the HP was dissolved in a mixture of dimethylsulfoxide-*d*6 (Aladdin, China) and deuterated water (Aladdin, China) at a concentration of 10 mg/mL. The element composition in the HA and HP was analyzed using K-Alpha X-ray Photoelectron Spectroscopy (Thermo Scientific, USA). The absorbance of the HA and HP solutions was measured using a UV-2600 ultraviolet spectrophotometer (Shimadzu, Japan) in the range from 200 to 350 nm. The molecular weights of HA and HP were measured using a PL-GPC50 gel permeation chromatograph (Agilent, USA) by dissolving them in a 50 v/v% ethanol-water solution at a concentration of 2 mg/mL.

Solubility of HP

The HA and HP were added to 10 mL of deionized water at a concentration of 10 mg/mL, followed by stirring for 4 h at room temperature. The absorbance of solutions was measured at 400 nm using an ultraviolet spectrophotometer.

ARS complexation assay

The ARS working solution was prepared by dissolving ARS in PBS solution (pH = 7.4) to achieve a final concentration of 0.02 *wt*/*v*%. The HA, APBA, or HP (1 mg) was individually dissolved in 1 mL of PBS solutions and subsequently centrifuged to remove any undissolved material. Then, the ARS working solution was mixed separately with an equal volume of PBS, HA, APBA, and HP solutions. The change in color of the mixed solutions was recorded, and the absorbance was measured using a Shimadzu UV-2600 ultraviolet spectrophotometer within a range of 350 nm to 650 nm.

Morphology and size distribution of HP microparticles

The HP microparticles were collected by centrifugation and subsequently lyophilized for 2 days to obtain the dry microparticles. The dry HP microparticles were sprayed with gold for 60 s and examined using a Regulus 8100 scanning electron microscope (Hitachi, Japan). To determine the size distribution, the HP microparticles were

centrifuged and then resuspended in deionized water to achieve a concentration of 10 mg/mL. The size distribution of HP microparticles was measured using a Mastersizer 3000 laser particle analyzer (Malvern, UK).

Drug loading and release of luteolin-loaded HP (HPL) microparticles

Luteolin was selected as a model drug for the loading and releasing assays. Briefly, 10 mg of HP and 1 mg of luteolin were dissolved in 1 mL of 50 *v*/*v*% ethanol-water solution at 70 °C. Subsequently, the above solution was added dropwise to 10 mL of PBS solution at a rate of 50 μL/min, while maintaining a speed of 1200 rpm to obtain HPL microparticles. The HPL microparticles were separated by centrifugation and then resuspended in a PBS solution. The calibration curve was established by measuring the absorbance of luteolin in PBS solution at 348 nm across a concentration range from 5 to 50 μg/mL. The residual luteolin content in HPL microparticles was determined by measuring the amount of luteolin remaining in the PBS solution and the initially added luteolin. The drug encapsulation efficiency (EE) and loading capacity (LC) were calculated as follow:

EE (%) = amount of loaded luteolin / amount of added luteolin × 100%

LC (%) = amount of loaded luteolin / (amount of luteolin-loaded HPL microparticle) × 100%

The drug release kinetics of HPL microparticles were assessed in vitro using the dialysis membrane methods, as previously reported in studies [1-5]. Briefly, 1 mg of HPL microparticles were combined with 1 mL of PBS solution and then transferred into a dialysis tube (500 Da, Yuanye, China). The dialysis tube was immersed in PBS solution (10 mL, pH = 7.4) and incubated on a shaker (37 °C, 90 rpm). At days 0.5, 1, 2, 4, 6, 8, 14, 18, 21, 25, 30, and 35, 1 mL of incubation solution was collected, and an equal volume of fresh PBS solution was supplemented. The drug release kinetics of HPL microparticles were determined by measuring the incubation solutions with an ultraviolet spectrophotometer at 348 nm.

Chondrocyte cells isolation and culture

All animal experiments were conducted in accordance with the National Research Council's Guide for the Care and Use of Laboratory Animals. All animal procedures were approved by the Animal Ethics and Welfare Committee of the Southern University of Science and Technology (Approval number SUSTech-2020-188). Sprague-Dawley rats (six-week-old, female, weighing 200-250 g) were obtained from Guangdong Medical Laboratory Animal Center and used for isolating chondrocyte cells. The chondrocyte cells were isolated from the articular cartilage of rats under aseptic conditions, as previously reported [6]. The chondrocyte cells were cultured in DMEM medium supplemented with 1% penicillin-streptomycin and 10% fetal bovine serum. After reaching a confluence of 80%-90%, the chondrocyte cells were either passaged or used for subsequent experiments before their fifth passage.

Cytotoxicity assessment

Briefly, chondrocyte cells were seeded onto 96-well plates at a density of 5, 000 cells per well and cultured for 48 h. The medium was subsequently replaced with fresh complete medium, followed by the addition of solutions at one-tenth volume of the complete medium (Con group: PBS solution, HP group: 10 mg of HP microparticles suspended in 1 mL of PBS solution, or HPL group: 10 mg of HPL microparticles suspended in 1 mL of PBS solution). Chondrocyte cells were cultured for an additional 24 h, gently rinsed with PBS, and then incubated with 100 μL of cell counting kit-8 (CCK-8, Beyotime, China) working solution for 1 h. The samples were then measured using a microplate reader at 450 nm.

Construction of oxidative stress microenvironment in vitro

The in vitro oxidative stress microenvironment was induced by varying concentrations of hydrogen peroxide H_2O_2 [7]. In brief, chondrocyte cells were seeded onto a 96-well plate at a density of 5, 000 cells per well and cultured in an incubator for 48 h. Subsequently, the complete medium was replaced with a fresh complete medium containing various concentrations of H_2O_2 (0, 1.0, 1.5, 2.0, and 3.0 mM), respectively. After an additional incubation of 6 h, the cell activity was assessed using a CCK-8 assay, and the extracellular matrix (ECM) was stained with Alcian blue and Safranine-O according to the manufacturer's instructions.

Cell activity

The procedures for cell seeding and culture were the same as mentioned above in the intracellular ROS testing, with cells cultured in the normal medium serving as the NC group. Chondrocyte cells were cultured for an additional 6 h, gently rinsed with PBS, and then incubated with 200 μL of CCK-8 working solution for 1 h. Subsequently, 100 μL of incubation solution was transferred to a new 96-well plate and then measured using a microplate reader at 450 nm.

Alcian blue staining

The procedures for cell seeding and culture were the same as mentioned above in the intracellular ROS testing, with cells cultured in the normal medium serving as the NC group. After incubating for an additional 6 h, chondrocyte cells were fixed with 4% paraformaldehyde for 30 min and then stained with Alcian blue staining solution (pH = 2.5, Beyotime, China) for 12 h. Chondrocyte cells were rinsed three times with PBS solutions and then observed under a microscope.

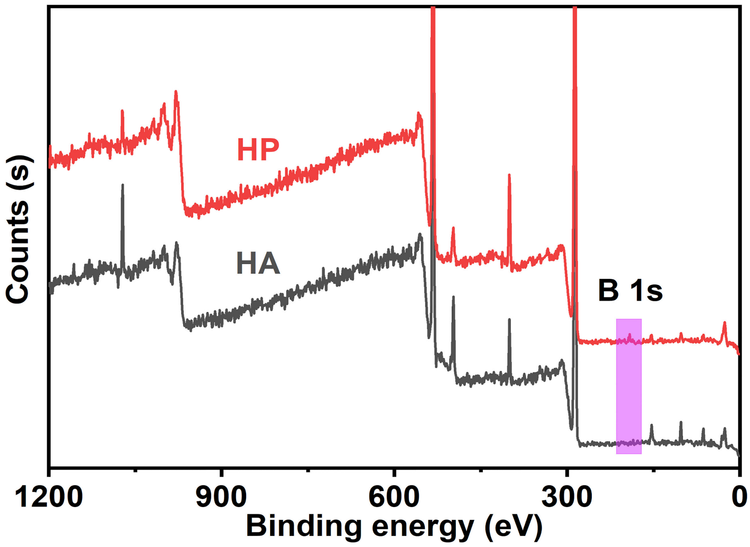

Fig. S1. XPS survey spectra of HA and HP.

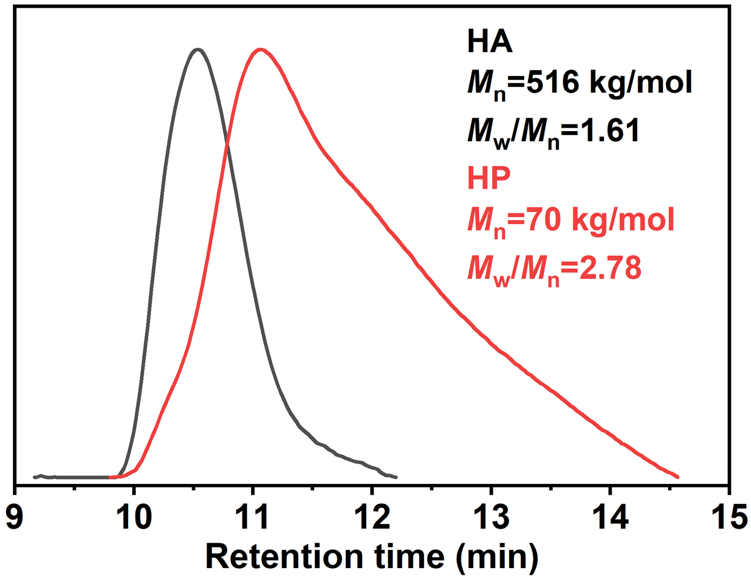

Fig. S2. GPC traces of HA and HP.

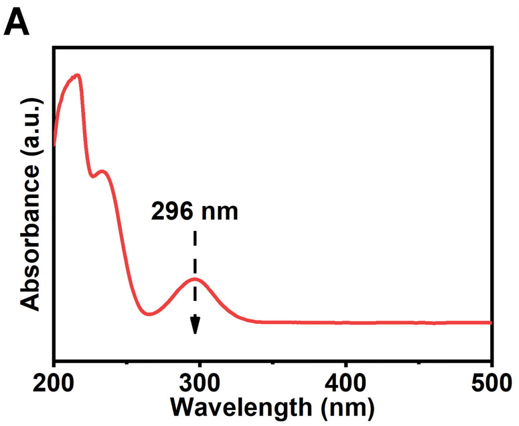

Fig. S3. The ultraviolet spectrum (a) and relevant standard curve (b) of APBA.

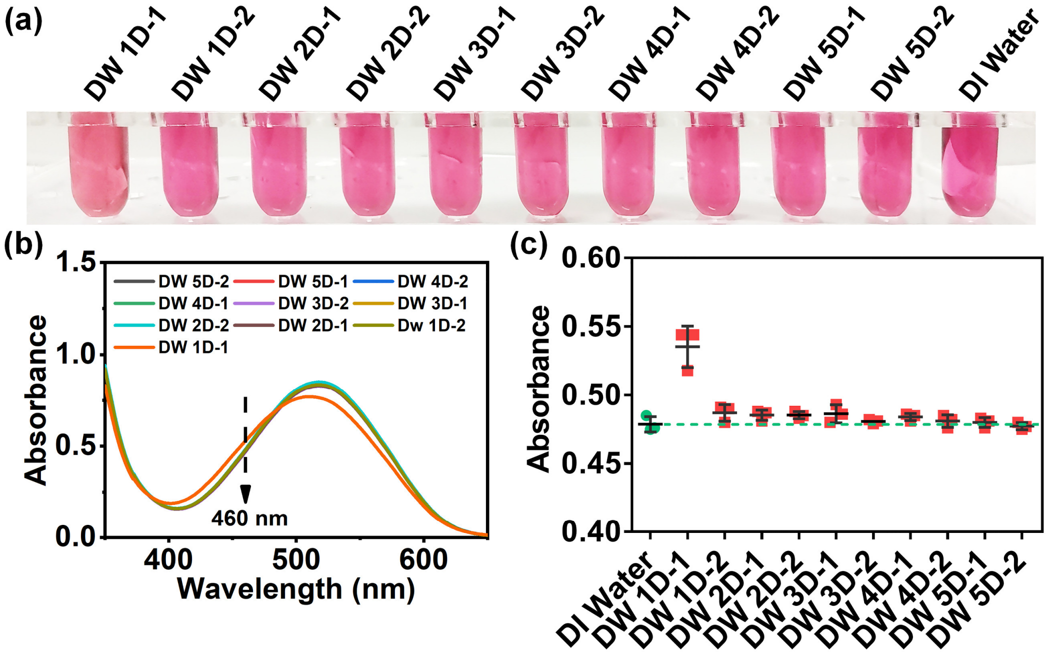

Fig. S4. (a) Image of color change, (b) ultraviolet spectra, and (c) relevant absorbance values at 460 nm of ARS solution mixed with dialysis water (DW) obtained from days 1 to 5. DW **X**D-**Y** represents the **Y**th collection of dialysis water on day **X**.

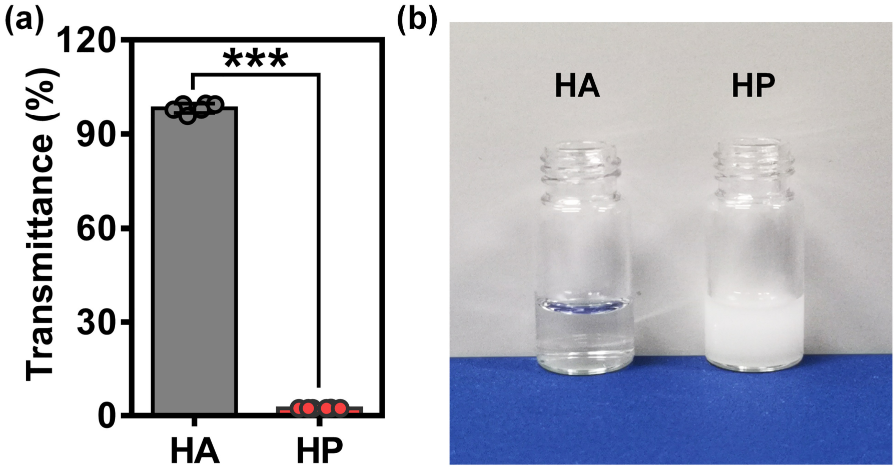

Fig. S5. The transmittance (a) and relevant images (b) of HA and HP dissolved in PBS solutions at a final concentration of 1 mg/mL.

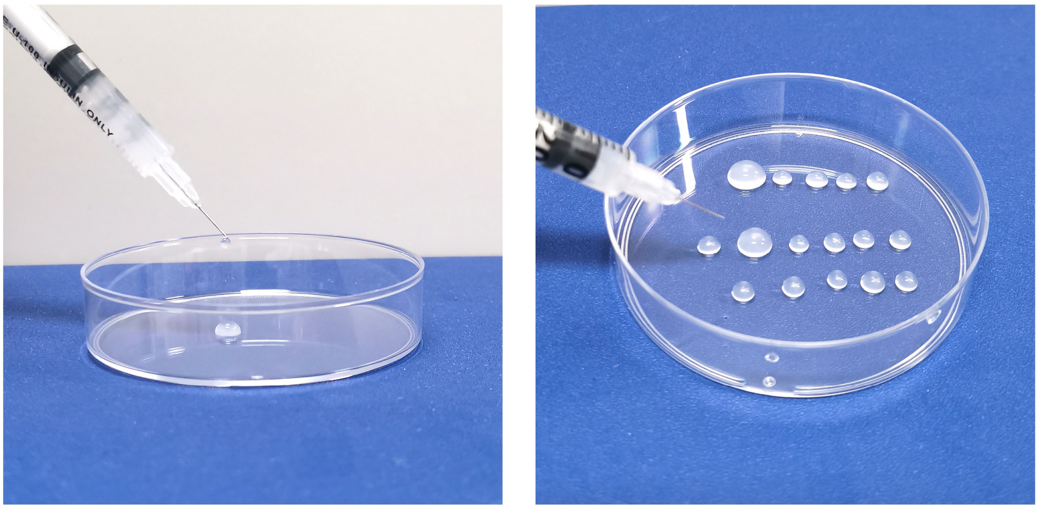

Fig. S6. The injection of HP microparticle solution through an insulin syringe needle.

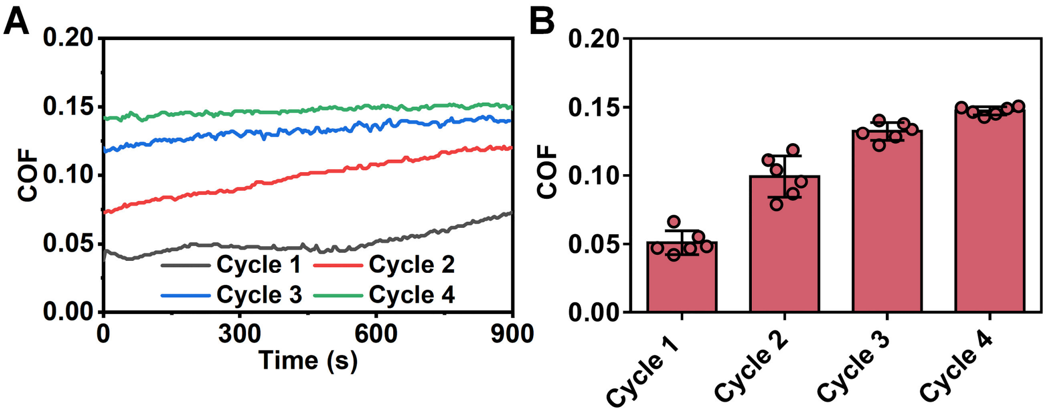

Fig. S7. (A) The COF-time curves and (B) COF values of HP microparticle solutions were measured for 4 repetitive cycles.

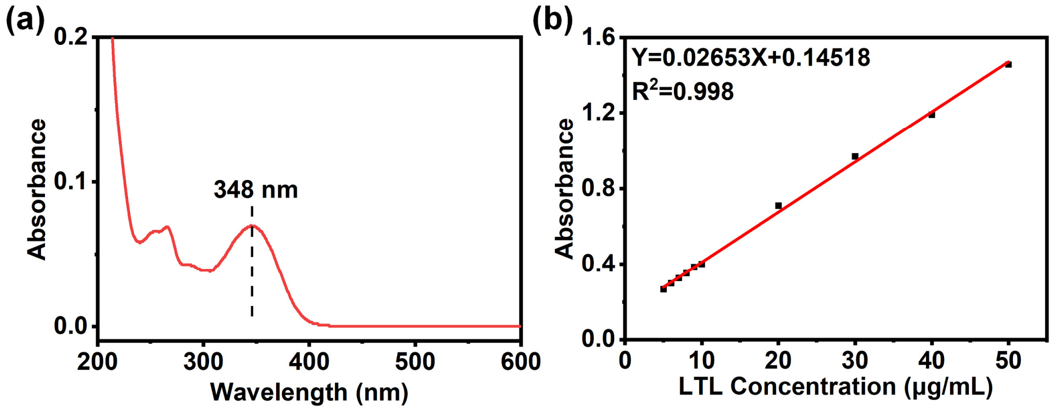

Fig. S8. The ultraviolet spectrum (a) and relevant standard curve (b) of luteolin.

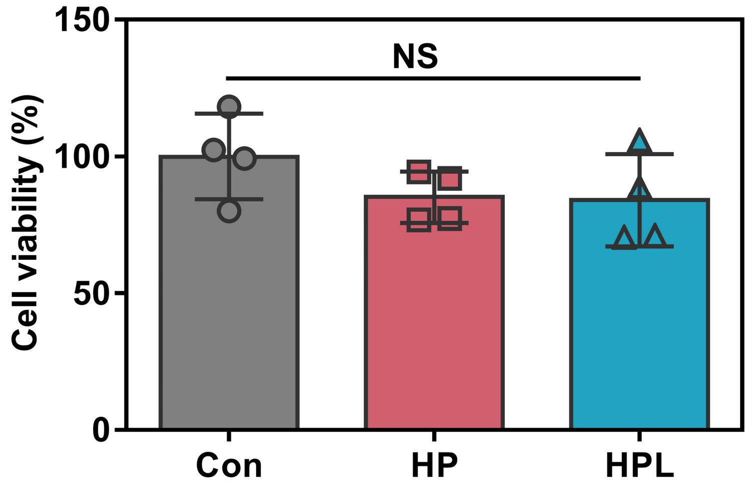

Fig. S9. The cell cytotoxicity of chondrocyte cells cultured with HP and HPL microparticles.

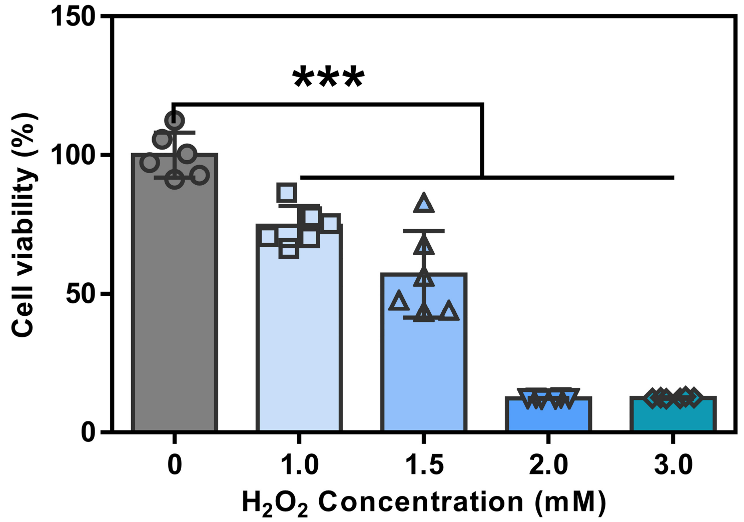

Fig. S10. The cell viability of chondrocyte cells cultured with various concentrations of H_2O_2 (n = 6).

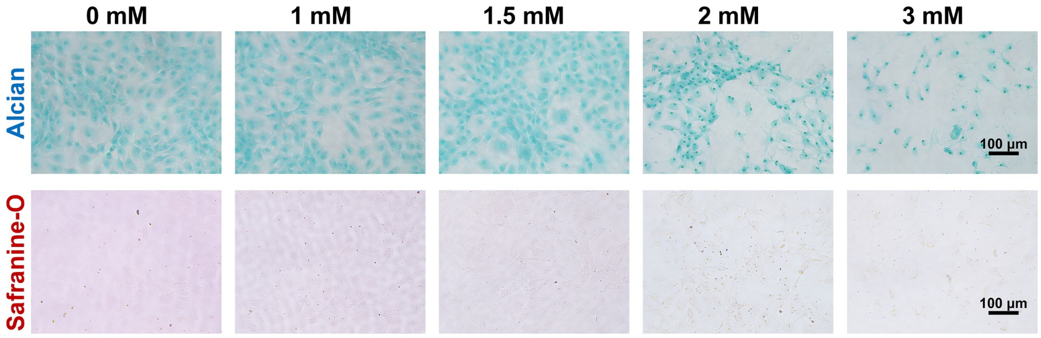

Fig. S11. The Alcian blue and Safranine-O staining of chondrocyte cells cultured with various concentrations of H_2O_2.

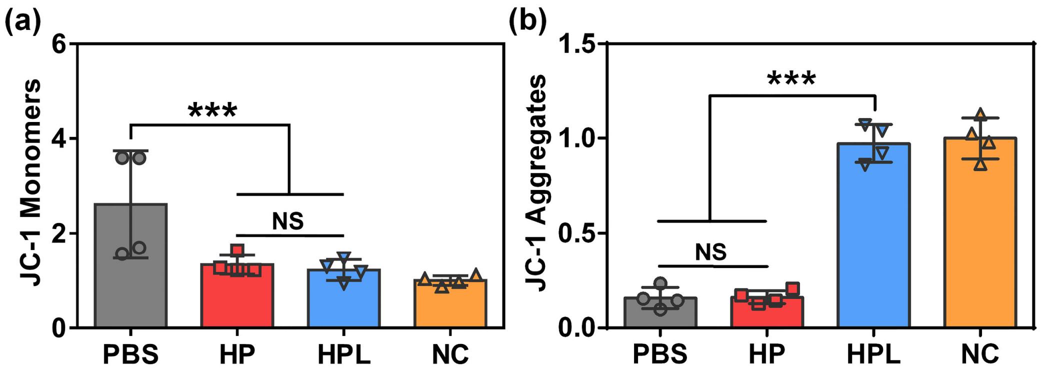

Fig. S12. The fluorescence intensity statistics of JC-1 monomers (a) and JC-1 aggregates (b).

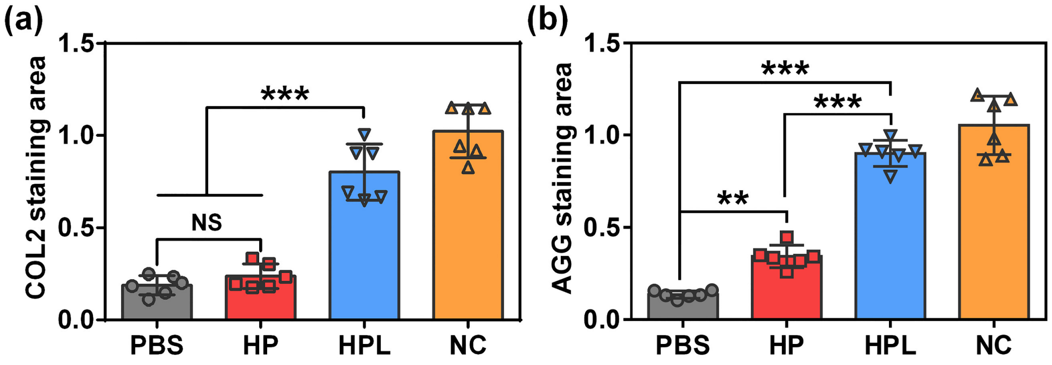

Fig. S13. The fluorescence staining area of (a) collagen type II (COL2) and (b) aggrecan (AGG).

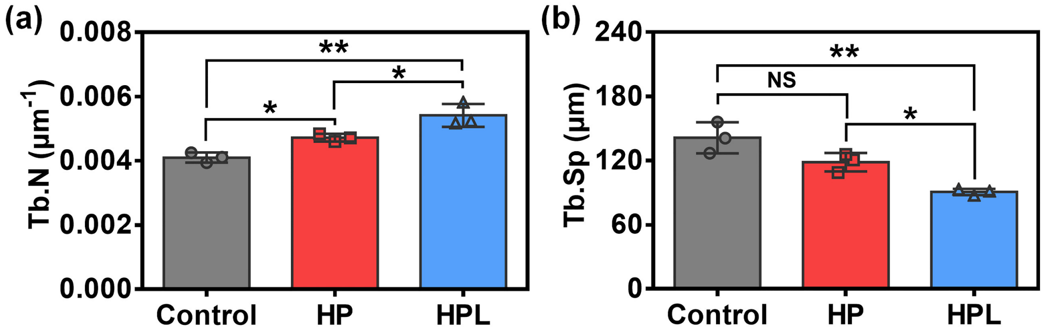

Fig. S14. Statistical analysis of (a) trabecular number (Tb.N) and (b) trabecular separation (Tb.Sp) based on Micro-CT analysis.

Table S1. The primer sequences of all genes used for RT-PCR analysis.

Gene Name	Direction	Primer Sequence
β-actin	Forward	AGATCAAGATCATTGCTCCTCCT
	Reverse	ACGCAGCTCAGTAACAGTC
COL2	Forward	ACGCTCAAGTCGCTGAACAACC
	Reverse	CCAGTAGTCTCCGCTCTTCCA
AGG	Forward	CCTGGACAAGTGCTATGCTGG
	Reverse	GCACCACTGACACACCTCGGAA
ADAMTS1	Forward	GGCTGATGTTGGAACTGTATGT
	Reverse	GATTGGGTTCTGCGGCTTGT
MMP13	Forward	AGCCCTATCCCTTGATGCCA
	Reverse	CTGAGCCTTTTCACCTCTTTTGG
IL-1β	Forward	ATGCCTCGTGCTGTCTGACC
	Reverse	TTTGTCGTTGCTTGTCTCTCCTTG
COX2	Forward	GATGACGAGCGACTGTTCCA
	Reverse	CAGCGGATGCCAGTGATAGA

Table S2. List of acronyms.

Acronyms	Synonyms
TMJ	temporomandibular joint
TMJOA	temporomandibular joint osteoarthritis
APBA	3-aminophenylboronic acid
HA	hyaluronic acid
HP	3-aminophenylboronic acid-modified hyaluronic acid
HPL	luteolin-loaded HP
DMTMM	4-(4,6-Dimethoxy-1,3,5-triazin-2-yl)-4-methyl morpholinium chloride
ARS	alizarin red S
DMEM	Dulbecco's modified eagle medium
S. aureus	Staphylococcus aureus
CCK-8	cell counting kit-8
ECM	extracellular matrix
ROS	reactive oxygen species
DCFH-DA	2',7'-dichlorofluorescin diacetate
JC-1	5,5',6,6'-tetrachloro-1,1',3,3'-tetraethyl-imidacarbocyanine
COL2	collagen type II
AGG	aggrecan
MMP13	matrix metalloproteinase
COX2	cyclooxygenase 2
Micro-CT	micro computed tomography
HE	Hematoxylin and Eosin
SF	Safranin-O-Fast green
FTIR	Fourier transform infrared
XPS	X-ray photoelectron spectroscopy
^{1}H NMR	proton nuclear magnetic resonance
SEM	scanning electron microscope
TEAC	Trolox equivalent antioxidant capacity
ABTS	2,2'-azino-bis (3-ethylbenzthiazoline-6-sulfonic acid
DPPH	2,2-diphenyl-1-picrylhydrazyl
UAC	unilateral anterior crossbite

Reference

1. Liu L, Xian Y, Wang W, Huang L, Fan J, Ma W, Li Y, Liu H, Yu J, Wu D. Meniscus-inspired self-lubricating and friction-responsive hydrogels for protecting articular cartilage and improving exercise. *ACS Nano*. 2023;17(23):24308-24319.
2. Lei Y, Wang X, Liao J, Shen J, Li Y, Cai Z, Hu N, Luo X, Cui W, Huang W. Shear-responsive boundary-lubricated hydrogels attenuate osteoarthritis. *Bioact Mater*. 2022;16:472-484.
3. Han Y, Yang J, Zhao W, Wang H, Sun Y, Chen Y, Luo J, Deng L, Xu X, Cui W, Zhang H. Biomimetic injectable hydrogel microspheres with enhanced lubrication and controllable drug release for the treatment of osteoarthritis. *Bioact Mater*. 2021;6(10):3596-3607.
4. Lin F, Wang Z, Xiang L, Deng L, Cui W. Charge‐guided micro/nano‐hydrogel microsphere for penetrating cartilage matrix. *Adv Funct Mater*. 2021;31(49):2107678.
5. Lei Y, Wang Y, Shen J, Cai Z, Zhao C, Chen H, Luo X, Hu N, Cui W, Huang W. Injectable hydrogel microspheres with self-renewable hydration layers alleviate osteoarthritis. *Sci Adv*. 2022;8(5):eabl6449.
6. Han Z, Bai L, Zhou J, Qian Y, Tang Y, Han Q, Zhang X, Zhang M, Yang X, Cui W, Hao Y. Nanofat functionalized injectable super-lubricating microfluidic microspheres for treatment of osteoarthritis. *Biomaterials*. 2022;285:121545.
7. Yu H, Teng Y, Ge J, Yang M, Xie H, Wu T, Yan Q, Jia M, Zhu Q, Shen Y, et al. Isoginkgetin-loaded reactive oxygen species scavenging nanoparticles ameliorate intervertebral disc degeneration via enhancing autophagy in nucleus pulposus cells. *J Nanobiotechnol*. 2023;21(1):99.
